# Microstructures and Interface Magnetic Moments in Mn_2_VAl/Fe Layered Films Showing Exchange Bias

**DOI:** 10.3390/nano11071723

**Published:** 2021-06-30

**Authors:** Takahide Kubota, Yusuke Shimada, Tomoki Tsuchiya, Tomoki Yoshikawa, Keita Ito, Yukiharu Takeda, Yuji Saitoh, Toyohiko J. Konno, Akio Kimura, Koki Takanashi

**Affiliations:** 1 Institute for Materials Research, Tohoku University, Sendai 980-8577, Japan; yshimada@imr.tohoku.ac.jp (Y.S.); keita.ito.e3@tohoku.ac.jp (K.I.); tjkonno@imr.tohoku.ac.jp (T.J.K.); koki@imr.tohoku.ac.jp (K.T.); 2 Center for Spintronics Research Network, Tohoku University, Sendai 980-8577, Japan; 3 Center for Science and Innovation in Spintronics, Core Research Cluster, Tohoku University, Sendai 980-8577, Japan; chat.luna81@gmail.com; 4 Graduate School of Science, Hiroshima University, Higashi-hiroshima 739-8526, Japan; tomoki-ysk@hiroshima-u.ac.jp (T.Y.); akiok@hiroshima-u.ac.jp (A.K.); 5 Materials Sciences Research Center, Japan Atomic Energy Agency, Hyogo 679-5148, Japan; ytakeda@spring8.or.jp (Y.T.); ysaitoh@spring8.or.jp (Y.S.); 6 Graduate School of Advanced Science and Engineering, Hiroshima University, Higashi-hiroshima 739-8526, Japan

**Keywords:** Heusler alloy, exchange bias, antiferromagnet, TEM, XMCD

## Abstract

Heusler alloys are a material class exhibiting various magnetic properties, including antiferromagnetism. A typical application of antiferromagnets is exchange bias that is a shift of the magnetization curve observed in a layered structure consisting of antiferromagnetic and ferromagnetic films. In this study, a layered sample consisting of a Heusler alloy, Mn${}_{2}$VAl and a ferromagnet, Fe, is selected as a material system exhibiting exchange bias. Although the fully ordered Mn${}_{2}$VAl is known as a ferrimagnet, with an optimum fabrication condition for the Mn${}_{2}$VAl layer, the Mn${}_{2}$VAl/Fe layered structure exhibits exchange bias. The appearance of the antiferromagnetic property in the Mn${}_{2}$VAl is remarkable; however, the details have been unclear. To clarify the microscopic aspects on the crystal structures and magnetic moments around the Mn${}_{2}$VAl/Fe interface, cross-sectional scanning transmission electron microscope (STEM) observation, and synchrotron soft X-ray magnetic circular dichroism (XMCD) measurements were employed. The high-angle annular dark-field STEM images demonstrated clusters of Mn${}_{2}$VAl with the L2${}_{1}$ phase distributed only around the interface to the Fe layer in the sample showing the exchange bias. Furthermore, antiferromagnetic coupling between the Mn- and Fe-moments were observed in element-specific hysteresis loops measured using the XMCD. The locally ordered L2${}_{1}$ phase and antiferromagnetic Mn-moments in the Mn${}_{2}$VAl were suggested as important factors for the exchange bias.

## 1. Introduction

Heusler alloys have received growing attention for their rich physical properties, such as ferromagnetism, antiferromagnetism, half-metallic electronic structure, shape memory behavior, superconductivity, topological behavior, and so on [1,2]. Among them, antiferromagnetism has attracted much attention in a recently emerging research field of antiferromagnetic spintronics [3], as well as the conventionally studied exchange bias described by a shift of the magnetization curve in a layered structure consisting of antiferromagnetic and ferromagnetic materials [4,5]. Several antiferromagnetic Heusler compounds and related materials were studied for the exchange bias, such as Mn-(Pt or Fe)-Ga [6], Ni${}_{2}$MnAl [7,8,9], Ru${}_{2}$MnGe [10,11], Mn${}_{2}$VSi [12], Fe${}_{2}$VAl [13], Mn${}_{3}$Ga [14], Mn${}_{3}$Ge [15], and so on. Compared to other conventionally studied antiferromagnetic materials for exchange bias, such as Mn-Ir and Mn-Pt alloys, the noble-element-free Heusler compounds are also attractive in terms of the elemental strategy [16]. Among the antiferromagnetic Heusler alloys, the Mn-(Pt or Fe)-Ga alloys exhibited relatively large exchange bias [6]. However, these were in bulk form, which were unsuitable for practical spintronic device applications. In addition, the other materials were studied using film samples layered with ferromagnetic materials, and fully-ordered chemical phases were required for realizing the antiferromagnetism. For practical application, the disorder effect in a film sample is of interest.

**Figure 1:**
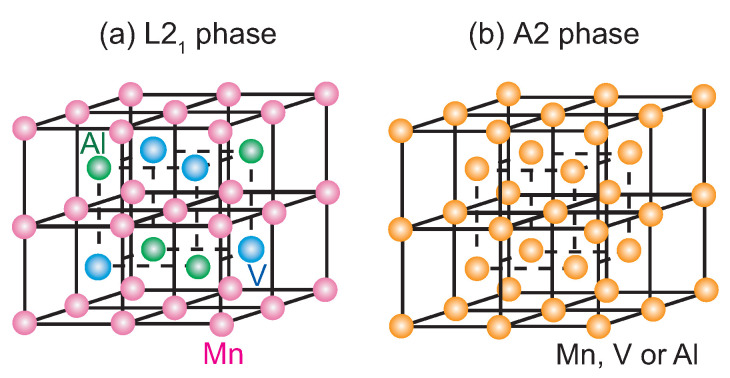
Schematic illustrations of the Heusler-type Mn${}_{2}$VAl compound for the (**a**) L2${}_{1}$ phase and (**b**) disordered A2 phase.

In the present study, Mn${}_{2}$VAl was selected as a material exhibiting exchange bias in layered structures with a ferromagnetic material. Figure 1 shows schematic crystal structures of Mn${}_{2}$VAl for the L2${}_{1}$ phase (Figure 1a) and A2 phase (Figure 1b). The crystal structure of the L2${}_{1}$ phase can be interpreted as four superimposed face-centered-cubic lattices where the Mn, V, and Al atoms are placed on the Wyckoff positions of 8c, 4b, and 4a, respectively. However, for the A2 phase, the atomic positions are chemically disordered. For the chemically ordered L2${}_{1}$ phase, the Mn${}_{2}$VAl is a ferrimagnet [17,18,19,20,21], which is also predicted as a half-metallic material with completely spin-polarized electrons at the Fermi level [22,23]. In addition, antiferromagnetism with a Néel temperature above 600 K was experimentally reported when the sample partially contained the disordered A2 phase in a bulk sample [24,25]. In film samples, a neutron diffraction study reported magnetic reflection suggesting the antiferromagnetism of a disordered Mn${}_{2}$VAl film at room temperature, and exchange bias was also observed in a disordered Mn${}_{2}$VAl/Fe layered structure [26]. Although the antiferromagnetism and exchange bias using the disordered Mn${}_{2}$VAl are remarkable, the origin remains unanswered. According to the *ab initio* calculation, Mn${}_{2}$VAl exhibits paramagnetism for the completely disordered A2 phase [27]. A possible factor causing the antiferromagnetism is the local chemical order of Mn${}_{2}$VAl, because in the study on the bulk sample [24,25], for example, very weak superlattice diffractions were observed in the X-ray diffraction pattern for the antiferromagnetic Mn${}_{2}$VAl. Thus, microscopic structural study, especially on the interface between the Mn${}_{2}$VAl and Fe layers of the layered film samples, is essential to discuss the mechanism of the antiferromagnetism and the exchange bias. Regarding the magnetic properties at the interface, soft X-ray magnetic circular dichroism (XMCD) is a powerful tool. The XMCD technique with the total electron yield (TEY) method can be a surface/interface-sensitive probe for the magnetic properties of layered film samples because of the limited transmittance of the soft X-ray beam, and the probing depth on the order of a few nanometers for the emitted electrons. In previous studies on the exchange bias, interface magnetic moments in antiferromagnets were evaluated using XMCD measurements for the layered samples, and uncompensated spin magnetic moments were detected in NiO [28], Pt-Mn [28], Ir-Mn [28,29], and Cr${}_{2}$O${}_{3}$ [30] antiferromagnetic films layered with ferromagnetic materials. However, no information on the microscopic structure and magnetic moment at the interface of the antiferromagnetic Mn${}_{2}$VAl has so far been provided. This has therefore motivated the unravelling of magnetic moments at the Mn${}_{2}$VAl/Fe interface, and the microscopic structure study using high-angle annular dark-field scanning transmission electron microscopy (HAADF-STEM) observation to get deeper insight into the exchange bias assisted by the Heusler alloy antiferromagnet.

## 2. Materials and Methods

Film samples were fabricated onto single-crystalline MgO (001) substrates using an ultra-high-vacuum (UHV) magnetron sputtering system. The stacking structure was a MgO substrate/Mn${}_{2}$VAl 100 nm/Fe $t_{\mathrm{Fe}}$/Ta 3 nm, where the Fe layer thickness ($t_{\mathrm{Fe}}$) was 0 or 3 nm. Prior to the layers’ deposition, the MgO substrates were annealed at 700 °C in the UHV chamber to obtain a clean MgO (001) surface, and subsequently, Mn${}_{2}$VAl/Fe layers were deposited. The deposition temperature, $T_{\mathrm{sub}}$, for the Mn${}_{2}$VAl layer was room temperature or 400 °C. The Fe layer was deposited at room temperature for all the samples to avoid the migration of Mn to the Fe layer. The Ta layer was also deposited at room temperature as a protection layer.

Four samples were prepared with the different $T_{\mathrm{sub}}$ and $t_{\mathrm{Fe}}$, as shown in Table 1. The magnetic and exchange bias properties have already been clarified in our previous study [26], as presented in Table 1. The microstructure was observed by conventional transmission electron microscopy (TEM) and high-angle annular dark-field scanning transmission electron microscopy (HAADF-STEM) with an acceleration voltage of 200 kV at room temperature using ARM-200F (JEOL, Ltd., Akishima, Japan). The incident direction of the electron beam was aligned to MgO[100]. Soft X-ray absorption spectroscopy (XAS) spectra and XMCD spectra were measured at BL23SU of SPring-8 [31]. The XAS and XMCD signals were obtained using the TEY method. A magnetic field was applied along the titled light propagation axis at an angle of 54.7° from the normal of the film plane. The measurement temperature was set at 100 K.

## 3. Results and Discussion

**Figure 2:**
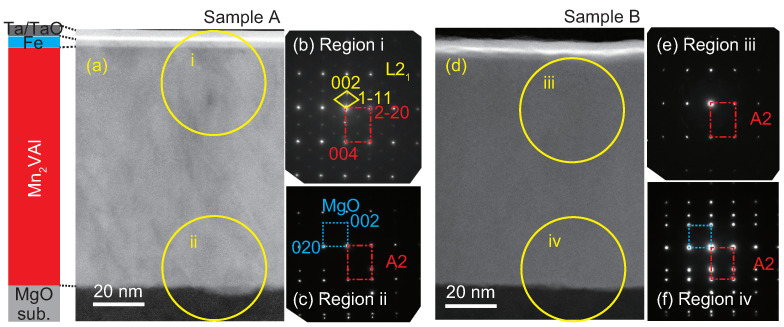
Cross-sectional low-magnification HAADF-STEM images for (**a**) Sample A and (**d**) B. Diffraction patterns around (**b**) the upper interface, region i, and (**c**) bottom interface, region ii of Sample A. Diffraction patterns around (**e**) the upper interface, region iii, and (**f**) the bottom interface region iv of Sample B. The yellow solid lines guide the superlattice diffractions for the L2${}_{1}$ phase in region i. The broken lines guide diffractions from the MgO substrates in regions ii and iv, and the dashed lines guide diffractions from the A2 phase in regions i to iv.

The wide-area cross-sectional TEM images are shown in Figure 2a,d of samples A and B, respectively. The layered structures are clearly confirmed in both images, and the interface between the Mn${}_{2}$VAl and Fe layers is smoother in Sample A than in Sample B. Electron diffraction images in the upper (bottom) region of samples A and B are also shown in Figure 2b,c,e,f, respectively. From the diffraction patterns, the epitaxial relationship between the MgO substrate and Mn${}_{2}$VAl layer is confirmed to be MgO[100](001) || Mn${}_{2}$VAl[110](001), which is consistent with the previous results obtained by X-ray and neutron diffraction techniques [26]. For the Mn${}_{2}$VAl layer, only the fundamental diffractions sets of 2 2 0 and 4 0 0 from the A2 phase are observed in Figure 2c,e,f. The superlattice diffractions set of 1 1 1 are confirmed in Figure 2b, which was taken in the upper region of Sample A for which the Mn${}_{2}$VAl layer was deposited at 400 °C.

**Figure 3:**
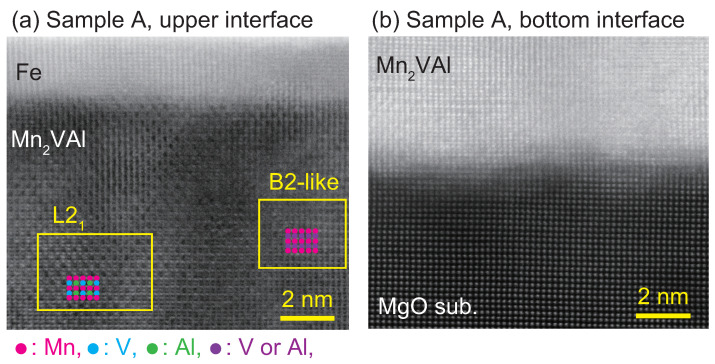
HAADF-STEM images of Sample A around (**a**) the upper Mn${}_{2}$VAl/Fe, and (**b**) the bottom MgO/Mn${}_{2}$VAl interfaces. Around the upper interface shown in (**a**), periodic contrast changes are observed for the Mn${}_{2}$VAl, which represent the L2${}_{1}$ phase and B2-like partially disordered phase. Around the bottom interface shown in (**b**), the entire area is the disordered A2 phase.

Figure 3 shows HAADF-STEM images at a high magnification for the interfaces between the Mn${}_{2}$VAl and Fe layer (upper region) of Sample A. The contrast periodically changes in the upper region, as shown in Figure 3, which corresponds to the L2${}_{1}$ phase or partially disordered B2 phase, in which the V and Al sites are mixed.

**Figure 4:**
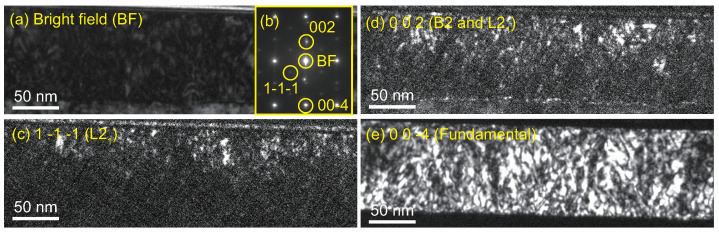
(**a**) Bright-field TEM image and (**b**) electron diffraction image. Corresponding dark-field images for (**c**) the 1 -1 -1 diffraction for the L2${}_{1}$ phase, (**d**) the 0 0 2 diffraction for the L2${}_{1}$ and B2 phases, and (**e**) the 0 0 -4, which is a fundamental diffraction of Sample A. The diffraction indexes are shown in (**b**).

To characterize the distribution of the ordered phases of L$2_{1}$ and B2, dark-field (DF) images were obtained for Sample A at a relatively low magnification. Figure 4a,b show a bright field image and a diffraction image at a relatively low magnification, for which corresponding DF images taken with the 1 -1 -1 (for the L2${}_{1}$ phase), 0 0 2 (for the L2${}_{1}$ and partially disordered B2 phases), and 0 0 -4 (the fundamental) diffractions are shown in Figure 4c–e, respectively. In the images, relatively small clusters of the L$2_{1}$ and B2 phases are distributed inside the matrix of the disordered A2 phase around the upper side of the Mn${}_{2}$VAl layer.

**Figure 5:**
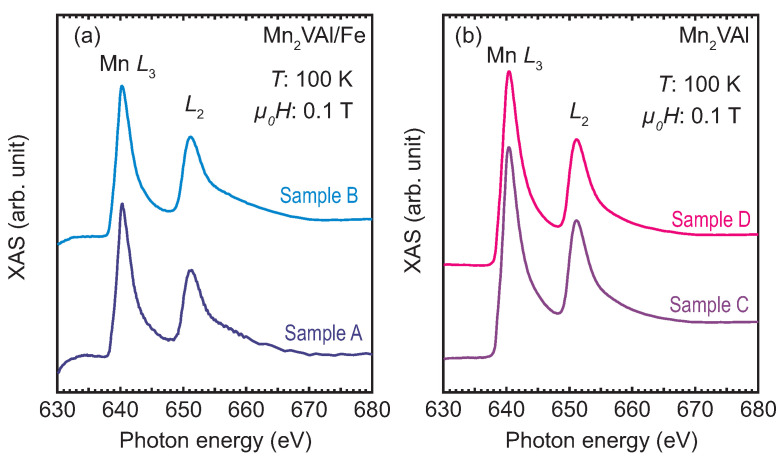
Soft X-ray absorption, XAS spectra around Mn $L_{3,2}$-absorption edges for (**a**) Mn${}_{2}$VAl/Fe layered and (**b**) Mn${}_{2}$VAl single-layer films. All spectra were measured at 100 K with an applied magnetic field (*H*) of 0.1 T. The XAS spectra in the panel (**a**) is expanded by a factor of 2.5 with respect to those in the panel (**b**).

XAS and XMCD measurements were conducted to probe site-specific magnetic moments at the Mn${}_{2}$VAl/Fe interface. Figure 5 shows the XAS spectra (=$\mu^{+}+\mu^{-}$) of all samples measured at a sample temperature of 100 K under an external magnetic field of 0.1 T applied along the line in parallel to the light propagation vector, which is tilted from the film surface at an angle of 54.7°. Here, $\mu^{+(-)}$ represents the absorption with positive (negative) helicity. All the XAS spectra shown in Figure 5 exhibit two main lines without fine structures at the Mn $L_{3,2}$-absorption edges. A spectral hump, which is expected to appear at approximately 645 eV for the ordered L2${}_{1}$ phase of Mn${}_{2}$VAl [19,21,32], is missing in the present data. It is consistent with the small volume fraction of the ordered phases in the samples, as evidenced by the present TEM images and supported by the previous X-ray diffraction experiment [26,27]. Having compared the spectral intensities of the samples with the Fe layer (samples A and B shown in Figure 5a) with those without the Fe layer (samples C and D shown in Figure 5b), we deduce that the intensities are smaller in samples A and B than those in samples C and D. This can be attributed to the relatively thick upper layers including the Fe layers on the Mn${}_{2}$VAl surfaces for samples A and B resulting in a smaller number of emitted electrons from the interface.

**Figure 6:**
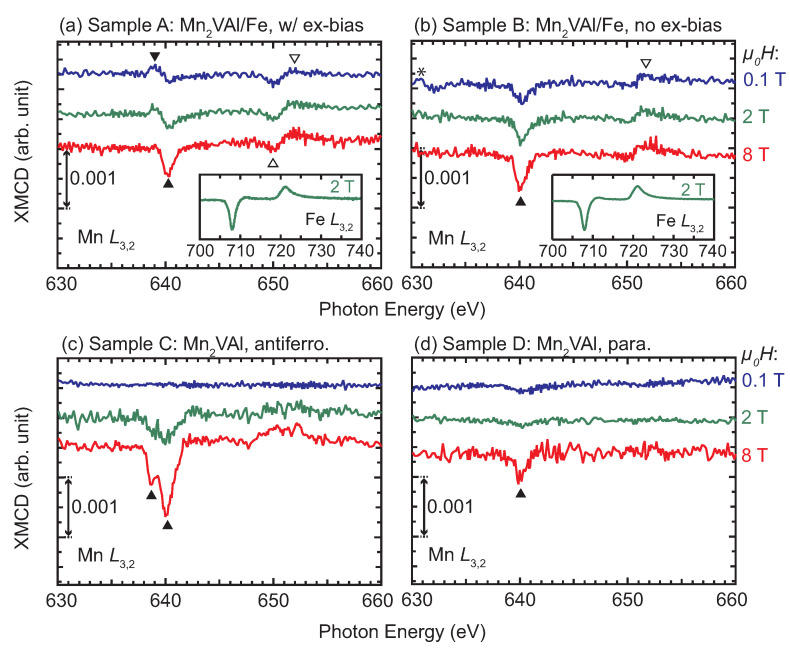
Soft X-ray magnetic circular dichroism (XMCD) spectra around the Mn $L_{3,2}$-absorption edges for the Mn${}_{2}$VAl/Fe layered and Mn${}_{2}$VAl single layer films measured at different *H*. (**a**) Sample A, (**b**) Sample B, (**c**) Sample C, and (**d**) Sample D. All spectra were measured at 100 K. Peaks observed around the $L_{3}$ ($L_{2}$)-absorption edge are marked by solid (open) triangles. A peak marked by ∗ is the experimental noise. The insets in panels (**a**,**b**) are the XMCD spectra around the Fe $L_{3,2}$-absorption edges for samples A and B, respectively.

**Figure 7:**
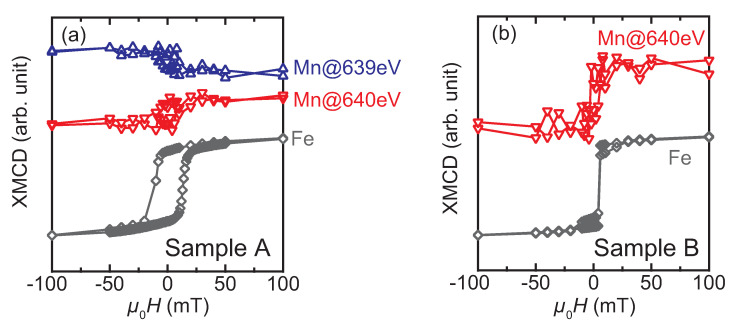
Element-specific MCD hysteresis loops, ESMHs, around the $L_{3}$-absorption edges of Mn and Fe for (**a**) Sample A, which is the Mn${}_{2}$VAl/Fe layered film showing exchange bias, and (**b**) Sample B, which is the other layered sample showing no exchange bias. The XMCD signals of Mn were arbitrarily scaled for visual comparison.

The XMCD spectra provides us with further information on the interface magnetic moments. Figure 6 shows the XMCD spectra measured at different external magnetic fields (*H*) for samples A to D. At $\mu_{0}H$ of 8 T, the finite XMCD amplitudes are observed for all the samples but with different spectral shapes to each other: In Sample A, a peak-dip feature and a dip-peak feature are observed around $L_{3}$- (the energy positions are marked by ▼ and ▲) and $L_{2}$-absorption (marked by the ▽ and △) edges, respectively, as shown in Figure 6a. We notice here that the feature near the $L_{3}$-edge is sensitive to the external magnetic field: the peak-to-dip amplitude remains unchanged at 2 T. At 8 T, the dip at 640 eV is deepened, whereas the peak at 639 eV is slightly shrunk. However, no significant variation with *H* is found for the dip-peak feature around the $L_{2}$-edge (∼650 and 651 eV). The XMCD spectrum of Sample B exhibits a dip at 640 eV and a dip-peak feature at 650–651 eV regardless of *H*. The dip at 640 eV grows with increasing *H*, whereas the dip-to-peak amplitude at the $L_{2}$ edge is insensitive to *H*. Because the positive peak at 639 eV is observed only in Sample A, it may be considered to be responsible for the exchange bias. The XMCD spectra around the $L_{3,2}$ absorption edges of Fe are also shown in the insets of Figure 6a,b, in which a dip (peak) is observed at 708 (721) eV. The spectral shapes of the samples A and B are similar, and resemble those of pure Fe, Heusler alloys containing Fe, and Fe-nitrides [33,34,35,36,37]. The XMCD spectra of samples C and D exhibit different features from those of samples A and B: particularly, there is no remarkable difference (or it is negligibly small) in the XMCD signal at 0.1 T. In Sample C, a dip appears at 2 T and its amplitude grows with *H*. A double minimum feature is observed at 8 T. It should be noted that the dip feature at 639 eV for Sample C can be distinguished from the peak for Sample A at the same photon energy. The XMCD spectrum of Sample D is the most insensitive to the small *H* (≤2 T) among the four samples. The dip amplitude increases at 8 T, whereas it is quite small below 2 T. Element-specific XMCD hysteresis loops (ESMHs) were also measured to identify the magnetization process of the interface magnetic moments. Figure 7 shows the ESMHs for samples A and B. During the measurements, the photon energies were fixed at 640 eV and 708 eV for the main peaks of Mn and Fe XMCD, respectively. For Sample A, the ESMH loop was also obtained at 639 eV to follow the hysteresis of the sub peak observed in the XMCD spectra in Figure 6a. The maximum $\mu_{0}H$ was ±3 T for all ESMH measurements, and field regions around the coercivity (within ±100 mT) are displayed for samples A and B.

**Figure 8:**
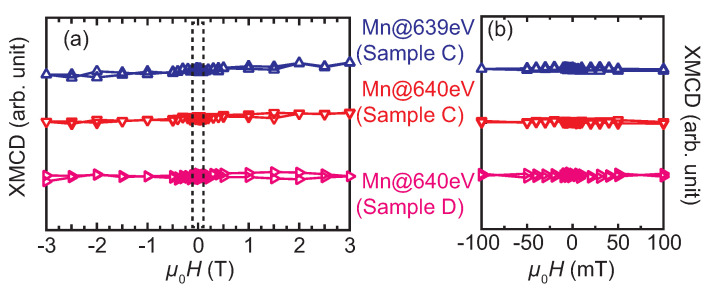
Element-specific MCD hysteresis loops, ESMHs, around the $L_{3}$-absorption edge of Mn for samples C and D, which are antiferromagnetic and paramagnetic Mn${}_{2}$VAl films, respectively. (**a**) Full-field range. (**b**) Expanded range surrounded by a broken square in (**a**).

Regarding Sample A, both ESMHs of the Mn XMCD measured at 639 and 640 eV exhibit hysteresis with different signs (Figure 7a): The sign of Mn-ESMH measured at 639 eV is opposite to that of Fe, which indicates that the Mn-moment and Fe-moment couple antiferromagnetically, whereas they are the same between the Mn-ESMH at 640 eV and Fe-ESMH representing ferromagnetic coupling. The antiferromagnetic coupling between the Mn and Fe atoms was previously reported in an Ir-Mn/Fe Sample Exhibiting exchange bias [38]. The ESMHs of Sample B at Mn (640 eV) and Fe edges also exhibit similar hysteresis showing the same signs as those of samples A (Figure 7b). The coercivity value is different between samples A and B, that is, the relatively large coercivity in Sample A is due to the exchange bias [26,27]. Here, no shift caused by the exchange bias is observed for the present ESMHs, because the expected shift is a few mT [26], which could not be detected due to the hysteresis of a superconducting magnet used for the measurements. In contrast, the ESMHs of samples C and D exhibit no hysteresis, as shown in Figure 8.

**Figure 9:**
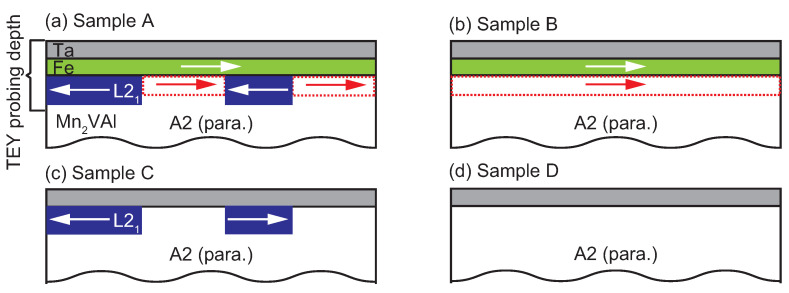
Schematic illustration of possible cases for the alignment of magnetic moments around the interface. For Samples A and C, the crystal grains showing the L2${}_{1}$ phase are distributed inside the A2 matrix. Here, $\mu_{0}H$ of 0.1 T is assumed in this Figure. In Samples (**a**) A and (**b**) B, the magnetic moments of Mn${}_{2}$VAl antiferromagnetically couple with that of Fe for the L2${}_{2}$ grains, whereas the induced moments in the A2 phase couple ferromagnetically. (**c**) In Sample C, although spontaneous moments occur inside the L2${}_{1}$ grain, those possibly align antiferromagneticaly, which results in no XMCD at 0.1 T. (**d**) In Sample D, no spontaneous magnetization occurs.

Two factors are considered here as origins of the XMCD signals, that is, (a) the spontaneous magnetization that is characteristic of the ferro- or ferri-magnetic order, and (b) the magnetic-field-induced magnetization, which is typically observed for paramagnetic samples and antiferromagnets under sufficiently large magnetic fields. Factor I plays a dominant contribution to the XMCD signals of samples A and B, whereas Factor II affects all samples; however, it is dominantly observed in samples C and D. The details are described as follows: Among the present samples, the XMCD for Sample D is simply caused by Factor II, in which the XMCD signal originates from the paramagnetic Mn-moments aligned by the relatively large external *H* [39,40,41,42]. The XMCD for Sample C is also caused by Factor II; however, the situation is different from that in Sample D. In Sample C, the clusters of the L2${}_{1}$ and B2 phases are considered to exist because of the analogy of the TEM images for Sample A shown in Figure 4, owing to the same deposition temperature for the Mn${}_{2}$VAl layer. Although Sample C shows no net magnetization because of the possible antiferromagnetism [9,24,25], the Mn-moments aligned by *H* exhibit XMCD with peak-splitting around the $L_{3}$-edge, which is similar to the fully-ordered Mn${}_{2}$VAl samples in previous studies [19,21,32]. For both cases of Samples C and D, no hysteresis is observed for the ESMHs shown in Figure 8, which implies that there is no spontaneous magnetization in the samples. The possible situations are schematically depicted in Figure 9c,d for Samples C and D, respectively.

**Figure 10:**
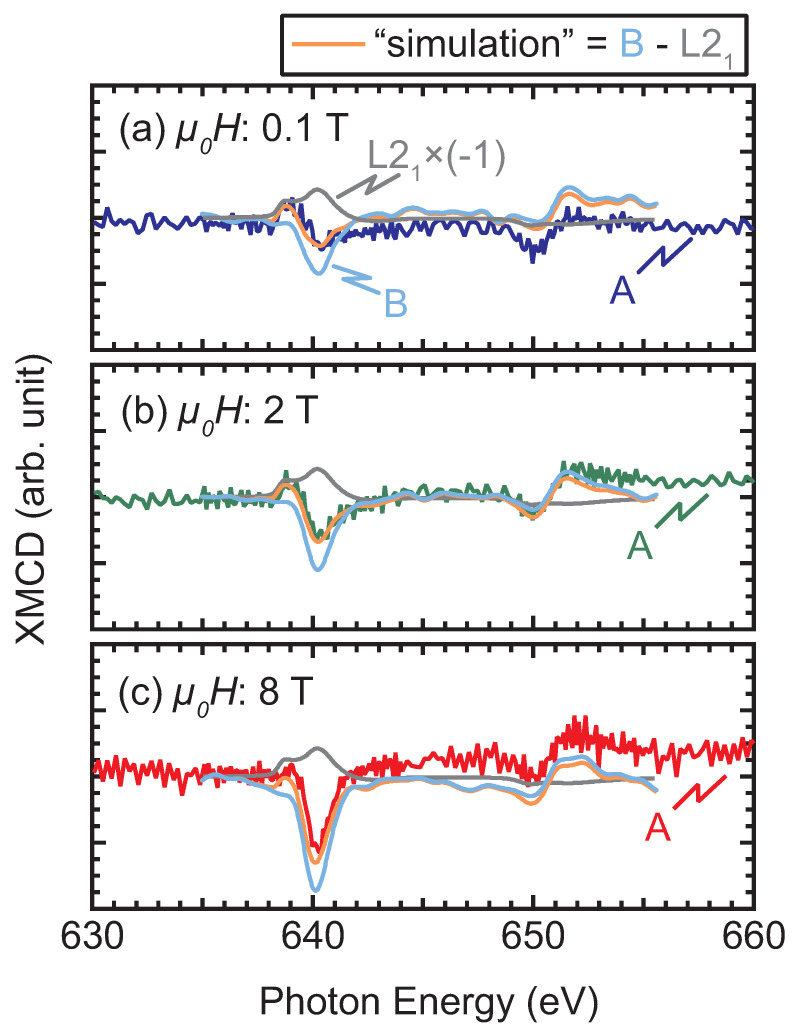
Decomposition of XMCD spectra of Sample A (annotated as “A”) using those of L2${}_{1}$- Mn${}_{2}$VAl and Sample B (annotated as “L2${}_{1}$” and “B”, respectively). The spectrum of L2${}_{1}$-Mn${}_{2}$VAl was obtained from the Ref. [32] with permission from American Phyical Society, 2018, in which a magnetic field of 2 T was applied for the measurement. The blue, green, and red spectra in panels (**a**–**c**), respectively, are the original data of Sample A shown in Figure 6a. The spectra of Sample B in each panel were smoothed and multiplied by a factor of 1.2 from the results shown in Figure 6b.

However, for the other cases of Samples A and B, finite XMCD signals at low $\mu_{0}H$ of 0.1 T indicate spontaneous magnetization, with Factor I as an origin: For the case of Sample B, the alignment of the Mn magnetic moments in the Mn${}_{2}$VAl layer is possibly assisted by the Fe layer. The moments at the interface of the A2-Mn${}_{2}$VAl ferromagnetically couple with the Fe moments. The situation in Sample A is partly different from that in Sample B in which the Mn-moments originally possess the local order in the L2${}_{1}$ clusters. Thus, the Mn moments in the paramagnetic A2 region ferromagnetically couple with the Fe-moments, whereas the Mn moments in the L2${}_{1}$ region couple antiferromagnetically with the Fe-moments, as is observed in the different signs of Mn-ESMHs shown in Figure 7a. The possible alignments of the magnetic moments for Samples B and A are depicted in Figure 9a,b, respectively. Based on the scenario above, the XMCD spectra of Sample A can be decomposed using the XMCD spectrum of the fully-ordered Mn${}_{2}$VAl with the L2${}_{1}$ phase and that of Sample B, which is summarized in Figure 10: For the decomposition, an XMCD spectrum of a bulk Mn${}_{2}$VAl measured at $\mu_{0}H$ of 2 T in Ref. [32] was used to simulate all the spectra of Sample A measured at different $\mu_{0}H$. The spectrum was arbitrarily scaled to adjust the peak amplitude, and the scaling factor was the same for all three cases. All XMCD spectra of Sample B were smoothed and multiplied by a factor of 1.2 from the original data shown in Figure 6b. The “simulation” spectrum in each panel of Figure 10 was calculated by subtracting the “L2${}_{1}$” spectrum from the spectrum of Sample B. Although the original data and “simulation” do not quantitatively match because of a small drift component due to the small experimental signals, the peak-dip structures are qualitatively reproduced in the simulation spectra. From the decomposed spectra, the antiparallel coupling between the Fe moments and the Mn moments in the L2${}_{1}$ phase is maintained up to 8 T, in which a short-range order of the Mn moments is induced by the neighboring ferromagnetic Fe layer. This can be an origin of the exchange bias in the Mn${}_{2}$VAl/Fe system.

## 4. Conclusions

The microstructures and magnetic moments at the interface between the Heusler alloy Mn${}_{2}$VAl and Fe layers were evaluated using cross-sectional HAADF-STEM images and XMCD spectra, respectively. The non-uniform distribution of the L2${}_{1}$ phase was confirmed in the Mn${}_{2}$VAl/Fe layered Sample Exhibiting exchange bias, in which clusters of the L2${}_{1}$ phase were distributed near the interface. The antiferromagnetically coupled Mn-moments to the Fe-moments were observed by the XMCD measurement in the layered film Sample Exhibiting exchange bias, whereas only ferromagnetic coupling was induced in the layered sample showing no exchange bias. 

## Figures and Tables

**Table 1:** Sample identification: The deposition temperature ($T_{\mathrm{sub}}$) for the Mn${}_{2}$VAl layer, Fe layer thickness ($t_{\mathrm{Fe}}$), and magnetic properties. AF and para represent antiferromagnetic and paramagnetic, respectively, which have been discussed in [26].

Sample	$T_{\mathrm{sub}}$ for Mn${}_{2}$VAl	$t_{\mathrm{Fe}}$	AF or para	Exchange Bias
A	400 °C	3 nm	AF	Yes
B	room temp.	3 nm	para	none
C	400 °C	0	AF	-
D	room temp.	0	para	-

## Data Availability

The data presented in this study are available on a reasonable request from the corresponding author.
